# Letter to the Editor Re: Mental Health Support for the Current and Future Medical Professionals during Pandemics

**DOI:** 10.31662/jmaj.2021-0171

**Published:** 2021-12-28

**Authors:** Amin Al-Hussainy, Maaz A. Khan, Sofyan Al Shdefat, Amir Ahmed, Sameeullah Shahabi

**Affiliations:** 1 School of Clinical Medicine, University of Cambridge, Cambridge, United Kingdom

**Keywords:** mental health, mental health support, COVID-19, medical students, medical professionals

## To the Editor:

As final year medical students at the University of Cambridge, we read with great interest the article by Saeki and Shimato titled “Mental Health Support for the Current and Future Medical Professionals during Pandemics” ^(1)^. The authors highlight the impact of the COVID-19 pandemic on the mental health of medical professionals and its implications. The authors also warn of the impact of neglect toward the mental health of medical students. As medical students who have experienced clinical placements before, during, and after the incipience of the pandemic, we feel well placed to offer further thoughts on the effects of COVID-19.

The pandemic has impaired clinical exposure for students, hindering the development of clinical skills and competence, which, as the authors suggest, can cause students to feel an increased level of insecurity. We believe that this may contribute to feelings of imposter syndrome, levels of which were extremely prevalent even before the pandemic, ranging from 22% to 60% according to a recent scoping review ^(2)^. The majority of studies in this review found that feelings of imposter syndrome were associated with increased rates of anxiety, depression, and burnout ^(2)^. If this continues post-graduation it can have resultant adverse effects on the physician’s health, patient care, and health care cost ^(3)^.

Moreover, the authors illustrate the increased rate of absences in the National Health Service in the United Kingdom due to psychological problems during the COVID-19 pandemic, as well as the increased levels of psychological stress faced by medical students ^(1)^. As the cohort of students impacted by the pandemic enter the workforce, we anticipate a population of doctors that have increased rates of mental health problems from the start of their professional career. Rates of absence may therefore increase further, straining an already struggling system.

Given the above, we welcome the authors’ suggestions to help address the psychological needs of medical students and professionals, particularly the organization of reflection sessions. As something that our Medical School regularly schedules, we have found that they foster a sense of camaraderie and create a healthy environment for the discussion of our needs and feelings. Such sessions are also protective against imposter syndrome ^(2)^. We also suggest that doctors affected by the pandemic during their university studies should receive increased educational and pastoral support. This could include frequent meetings with educational supervisors (in the UK context) and more scheduled practical skills teaching to compensate for lost time.

## Article Information

### Conflicts of Interest

None

### Author Contributions

All authors conceived the idea and contributed toward the design. AAH and MAK wrote the manuscript. All authors critically reviewed the manuscript and provided final approval. All authors agree to be accountable for all aspects of the work.

### Approval by Institutional Review Board (IRB)

Not needed.
